# Supplementary material for: The complexity of multiple CRISPR arrays in strains with (co-occurring) CRISPR systems
Source: Microlife. 2026 Jan 15;7:uqaf042. doi: 10.1093/femsml/uqaf042 (PMC12814883; doi:10.1093/femsml/uqaf042)
Supplement: uqaf042_Supplemental_File [file uqaf042_supplemental_file.pdf]

Supplementary Material: The complexity of multiple CRISPR arrays in strains with (co-occurring) CRISPR systems

Axel Fehrenbach[1,2], Alexander Mitrofanov[3], Rolf Backofen[3,4], and Franz Baumdicker[1,2]

[1]Cluster of Excellence “Controlling Microbes to Fight Infections”, Mathematical and Computational Population Genetics, University of Tübingen, Tübingen, Germany
[2]Institute for Bioinformatics and Medical Informatics (IBMI), University of Tübingen, Tübingen, Germany
[3]Bioinformatics group, Department of Computer Science, University of Freiburg, Freiburg, Germany
[4]Signalling Research Centres BIOSS and CIBSS, University of Freiburg, Freiburg, Germany

Supplementary Table S1. CRISPR array per Cas locus statistics by Cas type, number of Cas loci with associated same repeat arrays and more. We show the average, median and maximum number of arrays per Cas locus and the total locus count for the respective Cas types. In the second half of the table, 'same repeat' gives the amount of Cas loci which have at least one associated array that has the same consensus repeat as another array within the same genome. Then, each of the following columns gives the counts of Cas loci with associated arrays that are in a genome with a 'partner' array (not necessarily belonging to the same Cas locus), that *additionally* to having the same consensus repeat has some spacer content similarity. '+ Sp. Overlap' additionally requires some amount of spacer overlap (not necessarily the same spacer order). '+ = Sp.' additionally requires exactly the same spacer content (not necessarily the same spacer order). '+ Sp. Subset' additionally requires the spacer content to be contained in the other array (not necessarily the same spacer order). '+ = Last Sp.' additionally requires arrays to have exactly the same last spacer (according to the predicted orientation). All of the counts ignore spacer orientation, i.e. spacer and reverse complement are considered the same. Note that the 'partner' arrays are only part of the same genome and not required to belong to the same Cas locus or Cas type. Orphan arrays are excluded for this table. Supplementary Table S2 gives the same statistics in terms of array numbers instead of Cas loci.

Cas Type	Average	Median	Max	Locus Count	Same Repeat	+ Sp. Overlap	+ = Sp.	+ Sp. subset	+ = Last Sp.
CAS-I-A	1.55	1	5	149	71	4	0	0	0
CAS-I-B	1.46	1	9	2981	638	61	4	5	8
CAS-I-C	1.37	1	12	2824	286	37	8	13	13
CAS-I-D	1.74	2	8	363	124	6	1	1	1
CAS-I-E	1.73	2	12	10859	2907	59	10	11	13
CAS-I-F	1.70	2	8	3021	824	30	1	2	5
CAS-I-U	1.41	1	8	469	59	2	0	1	0
CAS-II-A	1.07	1	5	2884	182	17	3	3	3
CAS-II-B	1.21	1	11	147	18	4	4	4	4
CAS-II-C	1.20	1	10	3007	150	38	27	27	27
CAS-III-A	1.97	2	11	2507	1257	423	15	146	195
CAS-III-B	1.72	1	10	930	283	22	1	1	1
CAS-III-C	1.58	1	4	57	21	1	0	0	0
CAS-III-D	1.82	1	9	560	171	9	0	2	3
CAS-III-E	2.00	2	2	1	1	0	0	0	0
CAS-IV-A	1.17	1	3	123	4	1	0	0	0
CAS-V-A	1.26	1	23	4189	511	61	15	17	24
CAS-V-B	1.13	1	4	192	8	2	1	1	1
CAS-V-F	1.39	1	6	1436	210	27	2	8	10
CAS-VI-A	1.45	1	4	20	2	0	0	0	0
CAS-VI-B	1.31	1	11	7658	797	48	16	18	25
CAS-VI-C	1.22	1	9	383	99	3	0	0	0
Overall	1.48	1.0	23	44760	8623	855	108	260	333

Supplementary Table S2. Locus and array count per Cas type, number of arrays with same repeat and more. Besides the count of Cas loci and array count of all Cas types, we show the number of arrays which have the same consensus repeat as at least one other array in the same genome (as is reported in terms of Cas loci in Supplementary Table S1). Moreover, we show the number of arrays with same consensus repeat and additional spacer similarity (with at least one other array in the same genome) described in the caption of Supplementary Table S1.

Cas Type	Locus Count	Array Count	Same Repeat	+ Sp. Overlap	+ = Sp.	+ Sp. Subset	+ = Last Sp.
CAS-I-A	149	231	113	8	0	0	0
CAS-I-B	2981	4364	1156	120	13	16	26
CAS-I-C	2824	3856	598	72	16	23	27
CAS-I-D	363	630	219	12	2	2	2
CAS-I-E	10859	18732	5931	116	14	15	24
CAS-I-F	3021	5148	1500	47	1	2	8
CAS-I-U	469	661	121	4	0	1	0
CAS-II-A	2884	3075	243	26	3	3	3
CAS-II-B	147	178	32	4	4	4	4
CAS-II-C	3007	3621	283	71	51	51	52
CAS-III-A	2507	4932	3162	1229	32	179	431
CAS-III-B	930	1601	530	32	1	1	1
CAS-III-C	57	90	31	1	0	0	0
CAS-III-D	560	1020	324	14	0	3	6
CAS-III-E	1	2	2	0	0	0	0
CAS-IV-A	123	144	7	2	0	0	0
CAS-V-A	4189	5277	709	79	21	23	32
CAS-V-B	192	217	14	5	4	4	4
CAS-V-F	1436	1998	309	48	3	9	17
CAS-VI-A	20	29	3	0	0	0	0
CAS-VI-B	7658	10020	1158	74	27	29	38
CAS-VI-C	383	466	112	3	0	0	0
Overall	44760	66292	16557	1967	192	365	675

Supplementary Table S3. CRISPR array length (the number of spacers in the array) statistics by Cas type.

Cas Type	Average	Median	Max	Array Count
CAS-I-A	51.49	42	409	231
CAS-I-B	29.13	18	587	4364
CAS-I-C	25.26	15	318	3856
CAS-I-D	42.93	25	385	630
CAS-I-E	16.78	11	246	18732
CAS-I-F	21.58	14.5	317	5148
CAS-I-U	25.06	15	587	661
CAS-II-A	15.93	12	158	3075
CAS-II-B	17.76	11	71	178
CAS-II-C	11.92	5	184	3621
CAS-III-A	15.71	14	276	4932
CAS-III-B	19.83	11	359	1601
CAS-III-C	27.39	17	131	90
CAS-III-D	19.89	9	233	1020
CAS-III-E	6.50	6.5	7	2
CAS-IV-A	10.51	10	69	144
CAS-V-A	8.24	4	204	5277
CAS-V-B	5.68	3	48	217
CAS-V-F	8.24	5	102	1998
CAS-VI-A	10.14	4	69	29
CAS-VI-B	6.83	4	300	10020
CAS-VI-C	5.35	3	66	466
Orphan	11.25	7	242	16689
Overall	15.11	9	587	82981

Supplementary Table S4. CRISPR array length (the number of spacers in the array) statistics by Cas type broken down by 'Cas is close' label. The 'Cas is close' is *yes* if the distance to its respective Cas locus is less than 10,000 bp and *no* otherwise. Orphan arrays are excluded for this table.

Cas Type	Cas is close	Average	Median	Max	Array Count
CAS-I-A	yes	52.11	39	192	169
	no	49.79	44.5	409	62
CAS-I-B	yes	32.03	22	587	3483
	no	17.68	7	374	881
CAS-I-C	yes	27.39	17	318	3293
	no	12.82	6	181	563
CAS-I-D	yes	48.71	33	164	484
	no	23.77	8	385	146
CAS-I-E	yes	18.10	12	246	14949
	no	11.54	8	184	3783
CAS-I-F	yes	22.50	16	317	4765
	no	10.07	6	97	383
CAS-I-U	yes	27.69	19	587	549
	no	12.14	4	146	112
CAS-II-A	yes	16.28	12	158	2969
	no	6.25	3	41	106
CAS-II-B	yes	20.47	13	71	131
	no	10.21	5	50	47
CAS-II-C	yes	17.41	12	184	2003
	no	5.12	4	145	1618
CAS-III-A	yes	15.98	15	276	4529
	no	12.66	7.0	165	403
CAS-III-B	yes	19.76	12	179	1265
	no	20.11	8	359	336
CAS-III-C	yes	29.51	17	131	63
	no	17.01	8	233	319
CAS-III-D	yes	21.20	11	198	701
	no	22.44	8	86	27
CAS-III-E	yes	6.5	6.5	7	2
	no	-	-	-	0
CAS-IV-A	yes	11.24	10	69	116
	no	7.5	4	25	28
CAS-V-A	yes	15.14	10	173	359
	no	7.74	4	204	4918
CAS-V-B	yes	14.35	7	48	17
	no	4.94	3	36	200
CAS-V-F	yes	11.93	7	102	103
	no	8.03	5	94	1895
CAS-VI-A	yes	8.00	4	23	5
	no	10.58	4	69	24
CAS-VI-B	yes	11.88	7	213	418
	no	6.61	4	300	9602
CAS-VI-C	yes	3.90	2.5	11	10
	no	5.38	3	66	456
Overall	yes	20.81	14	587	40383
	no	8.73	4	409	25909

Supplementary Table S5. Underlying table for the diagonal of Figure 1 in the main manuscript. The table shows the fold-change aloneness, expected and observed frequencies and counts for each Cas type as well as the p-value of the bionomial test. The last column shows significance, i.e. *p < 0.05, **p < 0.01, ***p < 0.001, ****p < 0.0001, if there are more than 50 observed or expected observations of a type.

Cas Type	Fold-change	Ratio	Obs. Freq	Exp. Freq	Obs. Count	Exp. Count	p-value	Signif.
CAS-I-A	-1.3374	0.7477	0.00083	0.00112	15	20.06	0.1533	
CAS-I-B	1.7150	1.7150	0.02914	0.01699	524	305.54	5.94E-14	****
CAS-I-C	1.4737	1.4737	0.02308	0.01566	415	281.61	<1.00E-15	****
CAS-I-D	1.3264	1.3264	0.00378	0.00285	68	51.27	0.0103	
CAS-I-E	2.5939	2.5939	0.33343	0.12854	5996	2311.57	<1.00E-15	****
CAS-I-F	3.3511	3.3511	0.07446	0.02222	1339	399.57	1.12E-12	****
CAS-I-U	1.3027	1.3027	0.00328	0.00252	59	45.29	0.0207	*
CAS-II-A	-1.1843	0.8444	0.00945	0.01120	170	201.33	0.0128	*
CAS-II-C	-1.7100	0.5848	0.01001	0.01712	180	307.81	<1.00E-15	****
CAS-III-A	-1.5883	0.6296	0.01462	0.02323	263	417.73	<1.00E-15	****
CAS-III-B	1.2164	1.2164	0.00823	0.00677	148	121.67	0.0088	**
CAS-III-D	1.1140	1.1140	0.00462	0.00414	83	74.51	0.1484	
CAS-V-A	-1.2758	0.7838	0.01946	0.02483	350	446.56	9.09E-07	****
CAS-V-B	-2.3441	0.4266	0.00050	0.00117	9	21.10	0.0026	
CAS-V-F	-1.7241	0.5800	0.00473	0.00815	85	146.54	2.17E-08	****
CAS-VI-B	-1.1444	0.8738	0.05210	0.05963	937	1072.37	7.43E-06	****
CAS-VI-C	-2.2904	0.4366	0.00117	0.00267	21	48.10	9.05E-06	****

Supplementary Table S6. Underlying table for all off-diagonal entries in Figure 1 in the main manuscript. The table shows the fold-change co-occurrence, expected and observed frequencies and counts for each Cas type as well as the p-value of the bionomial test. The last column shows significance, i.e. *p < 0.05, **p < 0.01, ***p < 0.001, ****p < 0.0001, if there are more than 50 observed or expected observations of a type pair. We omit pairs without any observed occurrences.

1. Cas Type	2. Cas Type	Fold-change	Ratio	Obs. Freq.	Exp. Freq.	Obs. Count	Exp. Count	p-value	Signif.
CAS-I-A	CAS-I-B	-2.0008	0.4998	5.56E-05	1.11E-04	1	2.00	0.4057	
CAS-I-A	CAS-I-C	-1.8443	0.5422	5.56E-05	1.03E-04	1	1.84	0.4498	
CAS-I-A	CAS-I-D	2.9786	2.9786	5.56E-05	1.87E-05	1	0.34	0.0452	
CAS-I-A	CAS-I-E	-7.5700	0.1321	1.11E-04	8.42E-04	2	15.14	3.46E-05	
CAS-I-A	CAS-III-A	-1.368	0.7311	1.11E-04	1.52E-04	2	2.74	0.4849	
CAS-I-A	CAS-III-B	11.2950	11.2950	5.00E-04	4.43E-05	9	0.80	1.38E-08	
CAS-I-A	CAS-III-D	22.5448	22.5448	6.12E-04	2.71E-05	11	0.49	7.40E-13	
CAS-I-A	CAS-V-A	1.0258	1.0258	1.67E-04	1.63E-04	3	2.92	0.3358	
CAS-I-A	CAS-V-F	2.0841	2.0841	1.11E-04	5.34E-05	2	0.96	0.0730	
CAS-I-A	CAS-VI-B	-1.0032	0.9968	3.89E-04	3.91E-04	7	7.023	0.5953	
CAS-I-B	CAS-I-C	1.1749	1.1749	1.84E-03	1.56E-03	33	28.09	0.1534	
CAS-I-B	CAS-I-D	1.3690	1.3690	3.89E-04	2.84E-04	7	5.11	0.1454	
CAS-I-B	CAS-I-E	-6.4061	0.1561	2.00E-03	1.28E-02	36	230.56	<1.00E-15	****
CAS-I-B	CAS-I-F	-9.9602	0.1004	2.22E-04	2.22E-03	4	39.85	5.53E-13	
CAS-I-B	CAS-I-U	2.4351	2.4351	6.12E-04	2.51E-04	11	4.52	0.0025	
CAS-I-B	CAS-II-A	1.3944	1.3944	1.56E-03	1.12E-03	28	20.08	0.0357	
CAS-I-B	CAS-II-C	1.1726	1.1726	2.00E-03	1.71E-03	36	30.70	0.1478	
CAS-I-B	CAS-III-A	1.2000	1.2000	2.78E-03	2.32E-03	50	41.67	0.0884	
CAS-I-B	CAS-III-B	5.1911	5.1911	3.50E-03	6.75E-04	63	12.14	8.11E-13	****
CAS-I-B	CAS-III-D	3.6332	3.6332	1.50E-03	4.13E-04	27	7.43	6.32E-09	
CAS-I-B	CAS-V-A	1.8634	1.8634	4.62E-03	2.48E-03	83	44.54	8.50E-08	****
CAS-I-B	CAS-V-B	-2.1044	0.4752	5.56E-05	1.17E-04	1	2.10	0.3785	
CAS-I-B	CAS-V-F	-1.0441	0.9578	7.79E-04	8.13E-04	14	14.62	0.5054	
CAS-I-B	CAS-VI-B	1.1967	1.1967	7.12E-03	5.95E-03	128	106.96	0.0207	*
CAS-I-B	CAS-VI-C	-1.5992	0.6253	1.67E-04	2.67E-04	3	4.80	0.2946	
CAS-I-C	CAS-I-D	-2.3563	0.4244	1.11E-04	2.62E-04	2	4.71	0.1510	
CAS-I-C	CAS-I-E	-4.1667	0.2400	2.84E-03	1.18E-02	51	212.50	<1.00E-15	****
CAS-I-C	CAS-I-F	1.6062	1.6062	3.28E-03	2.04E-03	59	36.73	0.0003	***
CAS-I-C	CAS-I-U	3.6028	3.6028	8.34E-04	2.32E-04	15	4.16	7.95E-06	
CAS-I-C	CAS-II-A	17.8843	17.8843	1.84E-02	1.03E-03	331	18.51	<1.00E-15	****
CAS-I-C	CAS-II-C	2.3678	2.3678	3.73E-03	1.57E-03	67	28.30	1.74E-10	****
CAS-I-C	CAS-III-A	-1.5361	0.6510	1.39E-03	2.14E-03	25	38.40	0.0142	
CAS-I-C	CAS-III-B	1.7880	1.7880	1.11E-03	6.22E-04	20	11.19	0.0056	
CAS-I-C	CAS-III-D	1.6060	1.6060	6.12E-04	3.81E-04	11	6.85	0.4468	
CAS-I-C	CAS-V-A	2.4116	2.4116	5.51E-03	2.28E-03	99	41.05	1.00E-13	****
CAS-I-C	CAS-V-F	-2.6940	0.3712	2.78E-04	7.49E-04	5	13.47	0.0079	
CAS-I-C	CAS-VI-A	7.9752	7.9752	1.11E-04	1.39E-05	2	0.25	0.0022	
CAS-I-C	CAS-VI-B	1.0448	1.0448	5.73E-03	5.48E-03	103	98.58	0.3054	
CAS-I-C	CAS-VI-C	-4.4209	0.2262	5.56E-05	2.46E-04	1	4.42	0.0651	
CAS-I-D	CAS-I-E	-38.7597	0.0258	5.56E-05	2.15E-03	1	38.69	<1.00E-15	
CAS-I-D	CAS-I-F	-6.6890	0.1495	5.56E-05	3.72E-04	1	6.69	0.0096	
CAS-I-D	CAS-II-C	-1.2878	0.7765	2.22E-04	2.86E-04	4	5.15	0.4143	
CAS-I-D	CAS-III-B	3.9288	3.9288	4.45E-04	1.13E-04	8	2.04	0.0003	
CAS-I-D	CAS-III-D	1.6040	1.6040	1.11E-04	6.93E-05	2	1.25	0.1308	
CAS-I-D	CAS-V-A	3.7466	3.7466	1.56E-03	4.16E-04	28	7.47	1.81E-09	
CAS-I-D	CAS-V-F	-2.4522	0.4078	5.56E-05	1.36E-04	1	2.45	0.2972	
CAS-I-D	CAS-VI-B	-1.7947	0.5572	5.56E-04	9.98E-04	10	17.95	0.0311	
CAS-I-D	CAS-VI-C	1.2423	1.2423	5.56E-05	4.48E-05	1	0.80	0.1930	
CAS-I-E	CAS-I-F	-2.4120	0.4146	6.95E-03	1.68E-02	125	301.52	<1.00E-15	****
CAS-I-E	CAS-I-U	-1.1786	0.8485	1.61E-03	1.90E-03	29	34.18	0.2146	
CAS-I-E	CAS-II-A	-2.0255	0.4937	4.17E-03	8.45E-03	75	151.92	2.92E-12	****
CAS-I-E	CAS-II-C	-4.8379	0.2067	2.67E-03	1.29E-02	48	232.27	<1.00E-15	****
CAS-I-E	CAS-III-A	-15.7729	0.0634	1.11E-03	1.75E-02	20	315.22	<1.00E-15	****
CAS-I-E	CAS-III-B	-3.2787	0.3050	1.56E-03	5.11E-03	28	91.82	1.00E-15	****
CAS-I-E	CAS-III-D	-2.8114	0.3557	1.11E-03	3.13E-03	20	56.22	2.31E-08	****
CAS-I-E	CAS-V-A	-1.2812	0.7805	1.46E-02	1.87E-02	263	336.98	1.39E-05	****
CAS-I-E	CAS-V-B	3.4549	3.4549	3.06E-03	8.85E-04	55	15.92	1.17E-12	****

1. Cas Type	2. Cas Type	Fold-change	Ratio	Obs. Freq.	Exp. Freq.	Obs. Count	Exp. Count	p-value	Signif.
CAS-I-E	CAS-V-F	-4.2535	0.2351	1.45E-03	6.15E-03	26	110.58	<1.00E-15	****
CAS-I-E	CAS-VI-A	-2.0585	0.4858	5.56E-05	1.14E-04	1	2.06	0.3904	
CAS-I-E	CAS-VI-B	-1.4049	0.7118	3.20E-02	4.50E-02	576	809.22	<1.00E-15	****
CAS-I-E	CAS-VI-C	-1.3961	0.7163	1.45E-03	2.02E-03	26	36.30	0.0464	
CAS-I-F	CAS-I-U	-2.9533	0.3386	1.11E-04	3.29E-04	2	5.91	0.0662	
CAS-I-F	CAS-II-C	-2.1133	0.4732	1.06E-03	2.23E-03	19	40.15	0.0002	
CAS-I-F	CAS-III-A	-9.0827	0.1101	3.34E-04	3.03E-03	6	54.49	1.00E-15	****
CAS-I-F	CAS-III-B	-2.6448	0.3781	3.34E-04	8.83E-04	6	15.87	0.0043	
CAS-I-F	CAS-III-D	-2.4295	0.4116	2.22E-04	5.40E-04	4	9.72	0.035	
CAS-I-F	CAS-V-A	2.0773	2.0773	6.73E-03	3.24E-03	121	58.25	<1.00E-15	****
CAS-I-F	CAS-V-B	1.8170	1.8170	2.78E-04	1.53E-04	5	2.75	0.0610	
CAS-I-F	CAS-V-F	-2.1240	0.4708	5.00E-04	1.06E-03	9	19.11	0.0083	
CAS-I-F	CAS-VI-B	1.3798	1.3798	1.07E-02	7.78E-03	193	139.88	8.00E-06	****
CAS-I-F	CAS-VI-C	4.6223	4.6223	1.61E-03	3.49E-04	29	6.27	7.12E-12	
CAS-I-U	CAS-II-A	-1.4883	0.6719	1.11E-04	1.66E-04	2	2.98	0.4284	
CAS-I-U	CAS-II-C	1.7579	1.7579	4.45E-04	2.53E-04	8	4.55	0.0426	
CAS-I-U	CAS-III-A	-2.0585	0.4858	1.67E-04	3.43E-04	3	6.18	0.1361	
CAS-I-U	CAS-III-B	3.3354	3.3354	3.36E-04	1.00E-04	6	1.80	0.0026	
CAS-I-U	CAS-III-D	3.6313	3.6313	2.22E-04	6.13E-05	4	1.10	0.0055	
CAS-I-U	CAS-V-A	1.2117	1.2117	4.45E-04	3.67E-04	8	6.60	0.2207	
CAS-I-U	CAS-V-F	-1.0833	0.9231	1.11E-04	1.20E-04	2	2.17	0.6317	
CAS-I-U	CAS-VI-B	1.1353	1.1353	1.00E-03	8.82E-04	18	15.85	0.2456	
CAS-II-A	CAS-II-C	-20.2429	0.0494	5.56E-05	1.12E-03	1	20.23	3.44E-08	
CAS-II-A	CAS-III-A	1.8577	1.8577	2.84E-03	1.53E-03	51	27.45	1.90E-05	****
CAS-II-A	CAS-III-B	-3.9984	0.2501	1.11E-04	4.45E-04	2	8.00	0.0138	
CAS-II-A	CAS-III-D	-1.2241	0.8169	2.22E-04	2.72E-04	4	4.90	0.4588	
CAS-II-A	CAS-V-A	4.8383	4.8383	7.90E-03	1.63E-03	142	29.35	<1.00E-15	****
CAS-II-A	CAS-V-F	1.3498	1.3498	7.23E-04	5.36E-04	13	9.63	0.1101	
CAS-II-A	CAS-VI-B	2.6533	2.6533	1.04E-02	3.92E-03	187	70.48	1.34E-12	****
CAS-II-A	CAS-VI-C	-1.5805	0.6327	1.11E-04	1.76E-04	2	3.16	0.3880	
CAS-II-C	CAS-III-A	5.5748	5.5748	1.30E-02	2.33E-03	234	41.97	8.26E-13	****
CAS-II-C	CAS-III-B	-1.2226	0.8179	5.56E-04	6.80E-04	10	12.23	0.3239	
CAS-II-C	CAS-III-D	2.29386	2.9386	1.22E-03	4.16E-04	22	7.49	3.99E-06	
CAS-II-C	CAS-V-A	1.0474	1.0474	2.61E-03	2.50E-03	47	44.87	0.3395	
CAS-II-C	CAS-V-F	1.3582	1.3582	1.11E-03	8.19E-04	20	14.72	0.0719	
CAS-II-C	CAS-VI-A	3.6482	3.6482	5.56E-05	1.52E-05	1	0.27	0.0314	
CAS-II-C	CAS-VI-B	1.6797	1.6797	1.00E-02	5.99E-03	181	107.76	3.92E-11	****
CAS-II-C	CAS-VI-C	-4.8333	0.2069	5.56E-05	2.69E-04	1	4.83	0.0464	
CAS-III-A	CAS-III-B	-41.1477	0.2411	2.22E-04	9.23E-04	4	16.59	0.0003	
CAS-III-A	CAS-III-D	-2.5400	0.3937	2.22E-04	5.65E-04	4	10.16	0.0263	
CAS-III-A	CAS-V-A	-3.0451	0.3284	1.11E-03	3.39E-03	20	60.90	1.02E-09	****
CAS-III-A	CAS-V-F	5.1543	5.1543	5.73E-03	1.11E-03	103	19.98	<1.00E-15	****
CAS-III-A	CAS-VI-B	3.2208	3.2208	2.62E-02	8.13E-03	471	146.24	6.54E-13	****
CAS-III-A	CAS-VI-C	-6.5574	0.1525	5.56E-05	3.65E-04	1	6.56	0.0107	
CAS-III-B	CAS-III-D	1.6896	1.6896	2.78E-04	1.65E-04	5	2.96	0.0799	
CAS-III-B	CAS-V-A	1.2967	1.2967	1.2E-03	9.86E-04	23	17.74	0.0899	
CAS-III-B	CAS-V-F	-1.1641	0.8590	2.78E-04	3.24E-04	5	5.82	0.4749	
CAS-III-B	CAS-VI-B	-4.7326	0.2113	5.00E-04	2.37E-03	9	42.59	4.92E-10	
CAS-III-B	CAS-VI-C	-1.9106	0.5234	5.56E-05	1.06E-04	1	1.91	0.4308	
CAS-III-D	CAS-V-A	1.1969	1.1969	7.23E-04	6.04E-04	13	10.86	0.2060	
CAS-III-D	CAS-V-F	1.1223	1.1223	2.22E-04	1.98E-04	4	3.56	0.2867	
CAS-III-D	CAS-VI-B	-2.3714	0.4217	6.12E-04	1.45E-03	11	26.08	0.0007	
CAS-V-A	CAS-V-B	4.8774	4.8774	8.34E-04	1.71E-04	15	3.08	1.71E-07	
CAS-V-A	CAS-V-F	1.7788	1.7788	2.11E-03	1.19E-03	38	21.36	0.0004	
CAS-V-A	CAS-VI-B	1.8615	1.8615	1.62E-02	8.69E-03	291	156.33	9.17E-14	****
CAS-V-A	CAS-VI-C	5.1342	5.1342	2.00E-03	3.90E-04	36	7.01	4.72E-13	
CAS-V-B	CAS-V-F	1.9817	1.9817	1.11E-04	5.61E-05	2	1.01	0.0820	
CAS-V-B	CAS-VI-B	1.3540	1.3540	5.56E-04	4.11E-04	10	7.39	0.1281	
CAS-V-F	CAS-VI-A	7.6630	7.6630	5.56E-05	7.26E-06	1	0.13	0.0078	
CAS-V-F	CAS-VI-B	-1.0059	0.9941	2.84E-03	2.85E-03	51	51.30	0.5204	
CAS-V-F	CAS-VI-C	-1.1505	0.8692	1.11E-04	1.28E-04	2	2.30	0.5958	
CAS-VI-A	CAS-VI-B	1.0472	1.0472	5.56E-05	5.31E-05	1	0.95	0.2477	

1. Cas Type	2. Cas Type	Fold-change	Ratio	Obs. Freq.	Exp. Freq.	Obs. Count	Exp. Count	p-value	Signif.
CAS-VI-B	CAS-VI-C	1.5441	1.5441	1.45E-03	9.36E-04	26	16.84	0.0136	

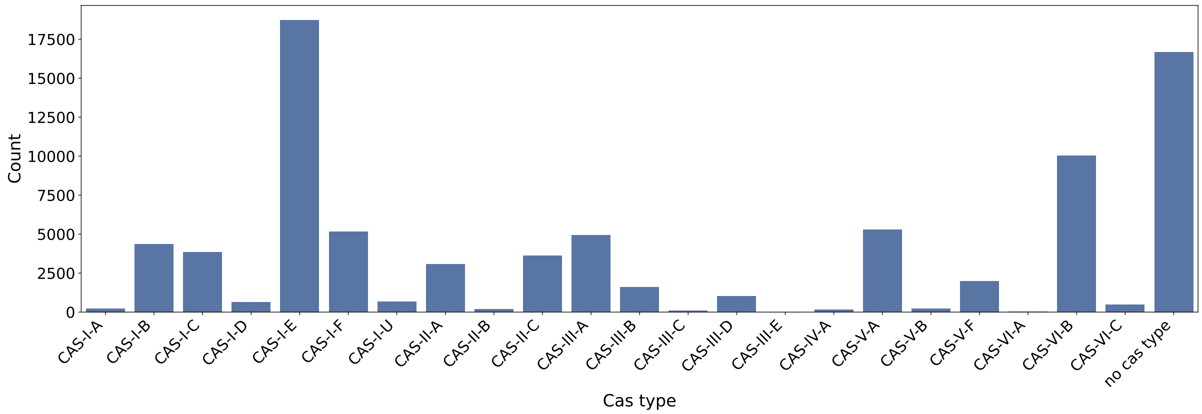

Supplementary Fig. S1. Distribution of Cas types across arrays. We show the distribution of Cas types of the arrays for the complete NCBI dataset.

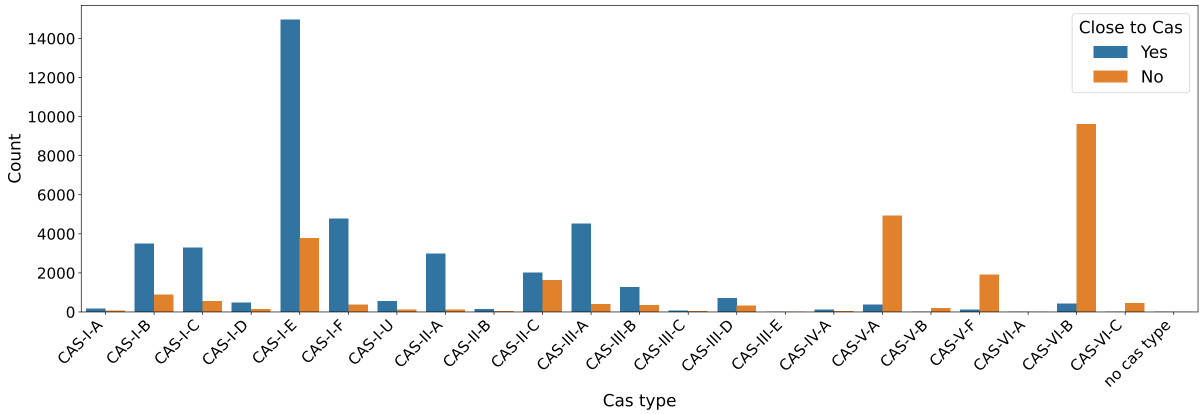

Supplementary Fig. S2. Distribution of Cas types across arrays with 'Cas is close' label. We break down the counts according to closeness of their Cas loci. We label a array to have a close Cas locus, if the Cas locus is less than 10000 bp removed from its Cas. Clearly, types V and VI tend to be far removed from their Cas loci and II-C shows a remarkable equal distribution between closeness and not.

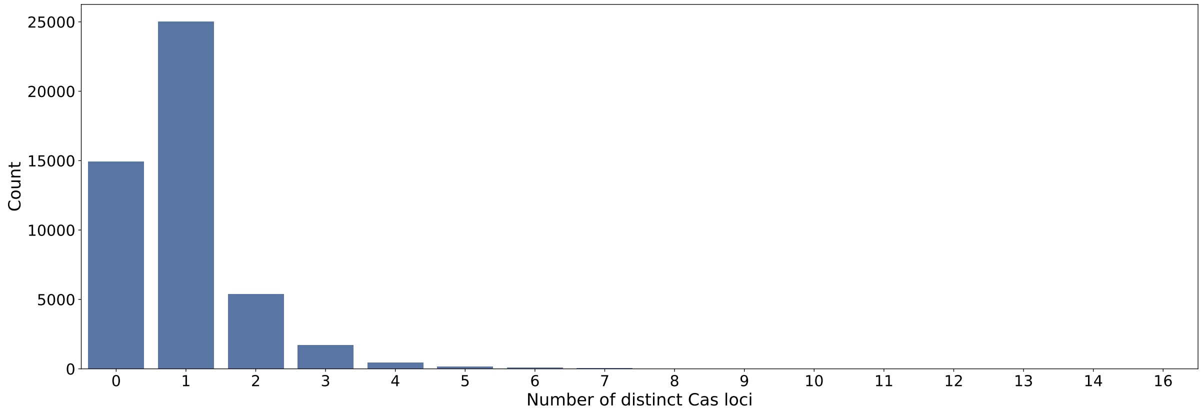

Supplementary Fig. S3. Distribution of number of distinct Cas loci within genomes. Here we show the distribution of the number of *distinct* Cas loci (not types!) per sample. 0 indicates that the array(s) within the genome are "orphan" arrays an do not contain *any* Cas locus. Since CRISPR arrays can only be orphans if no Cas locus exisits at all in the genome, there are no orphans in any other category than 0.

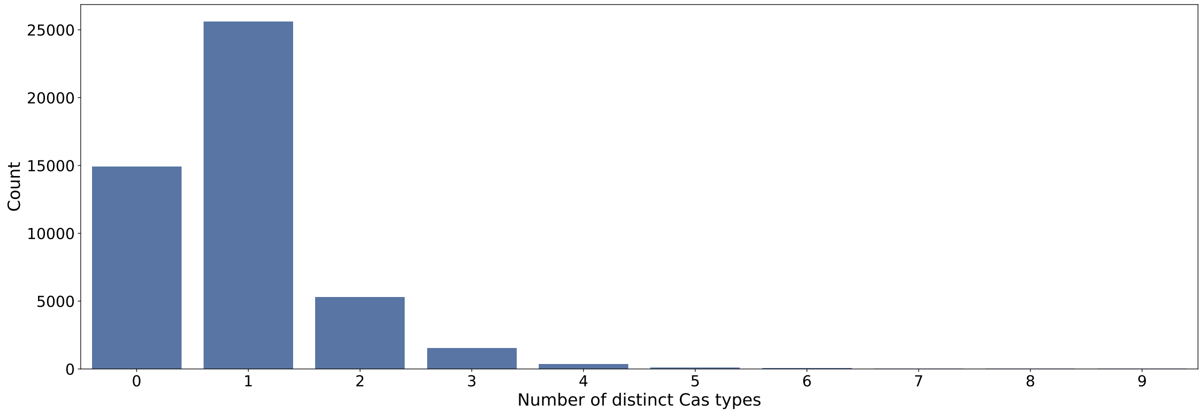

Supplementary Fig. S4. Distribution of number of distinct Cas types within genomes. Here a number of distinct Cas types of 0 means that the arrays within the genome are “orphan” arrays and do not contain *any* Cas loci. Since CRISPR arrays can only be orphans if no Cas locus exisits at all in the genome, there are no orphans in any other category than 0.

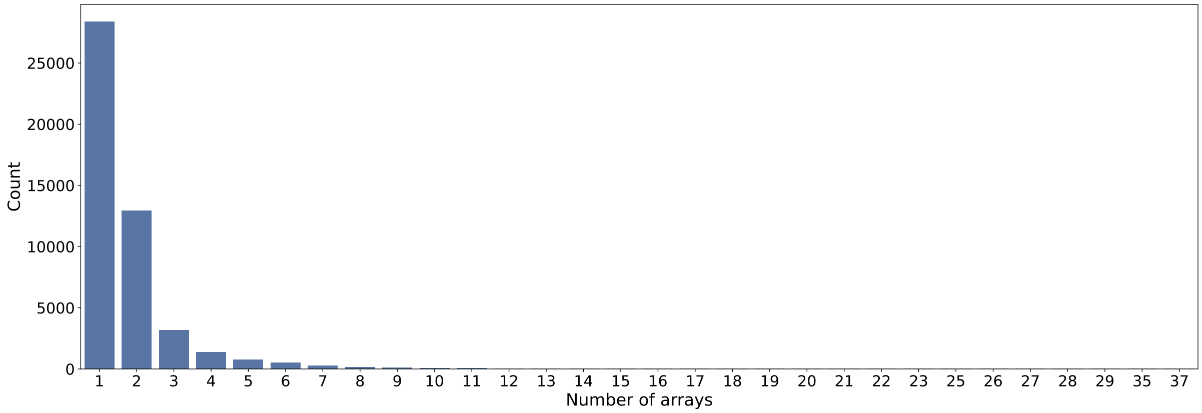

Supplementary Fig. S5. Distribution of number of arrays within genomes. We show all numbers on the x-axis for which at least one genome exists.

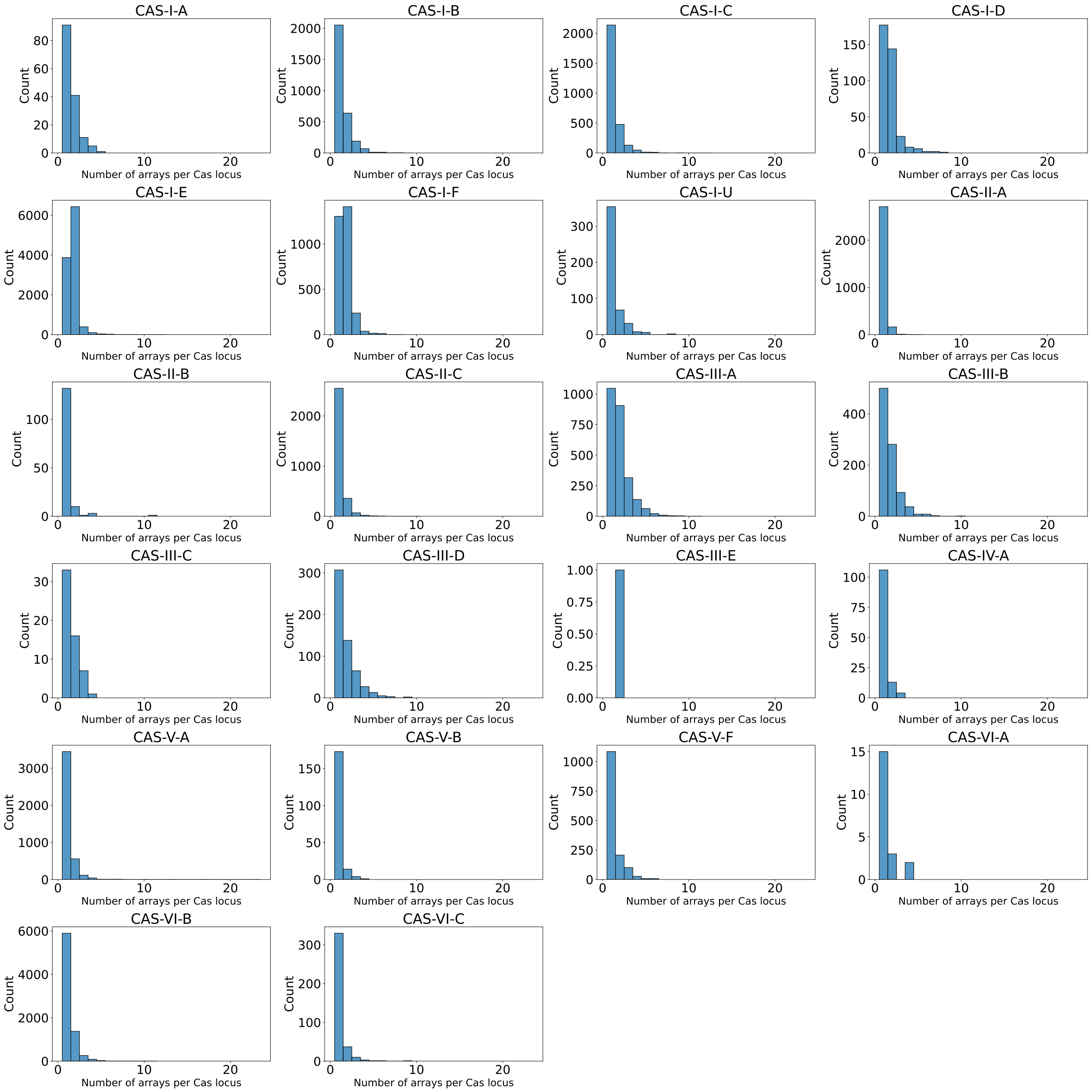

Supplementary Fig. S6. Distribution of number of arrays per Cas locus for different Cas types. We show distribution of the number of arrays belonging to a particular Cas locus. Type I-E, I-F and III-A tend to favor two arrays per Cas locus. Otherwise the distributions vary but are largely consistent.

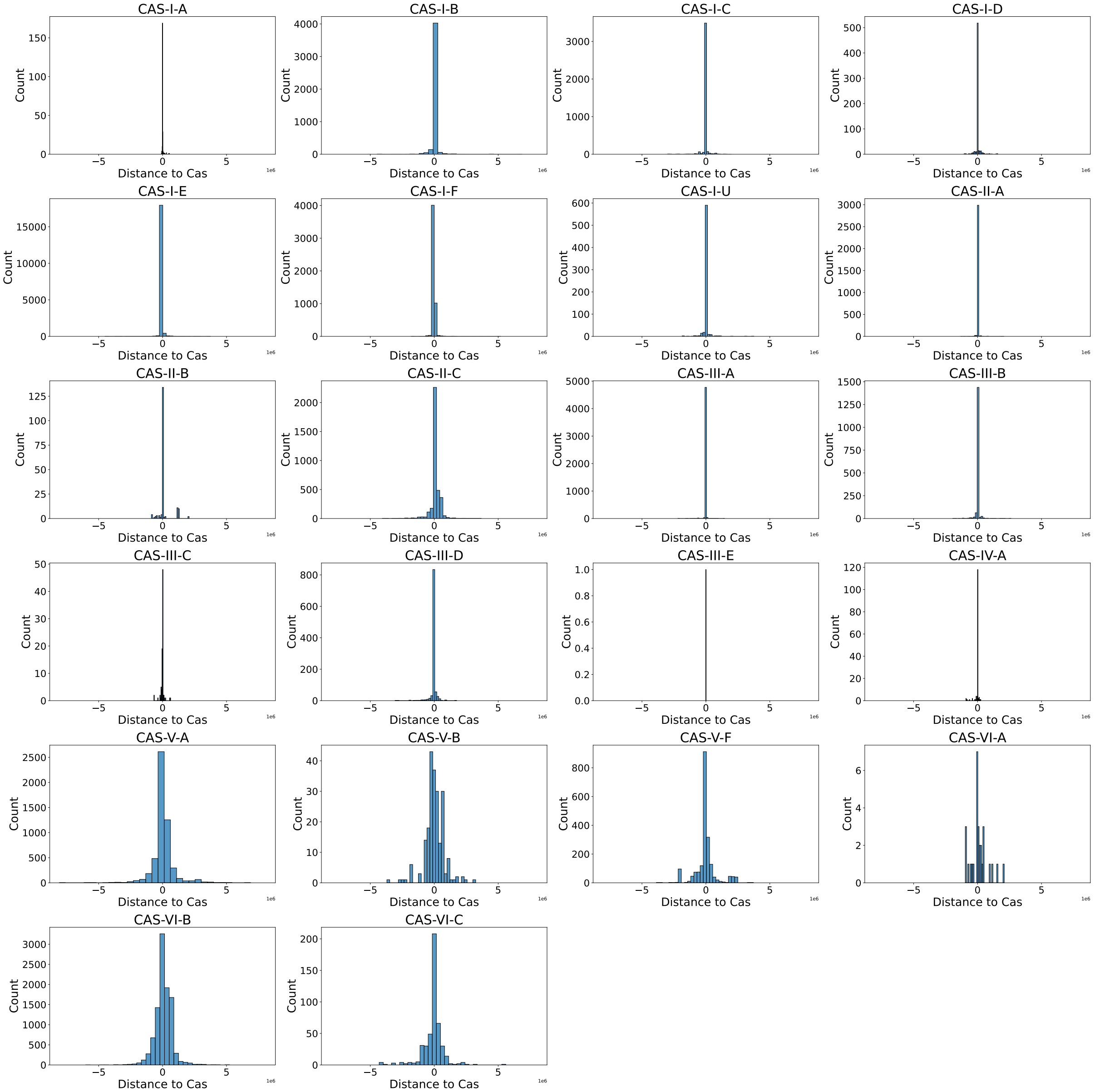

Supplementary Fig. S7. Directional distances of CRISPR arrays to their respective Cas locus. This figure clearly shows that types I, II and III are very concentrated around their Cas locus. Types V and VI however tend to be much further from their Cas loci. We excluded types with very few samples. Note that the x-axis is scaled in units of 10^6 base pairs (bp).

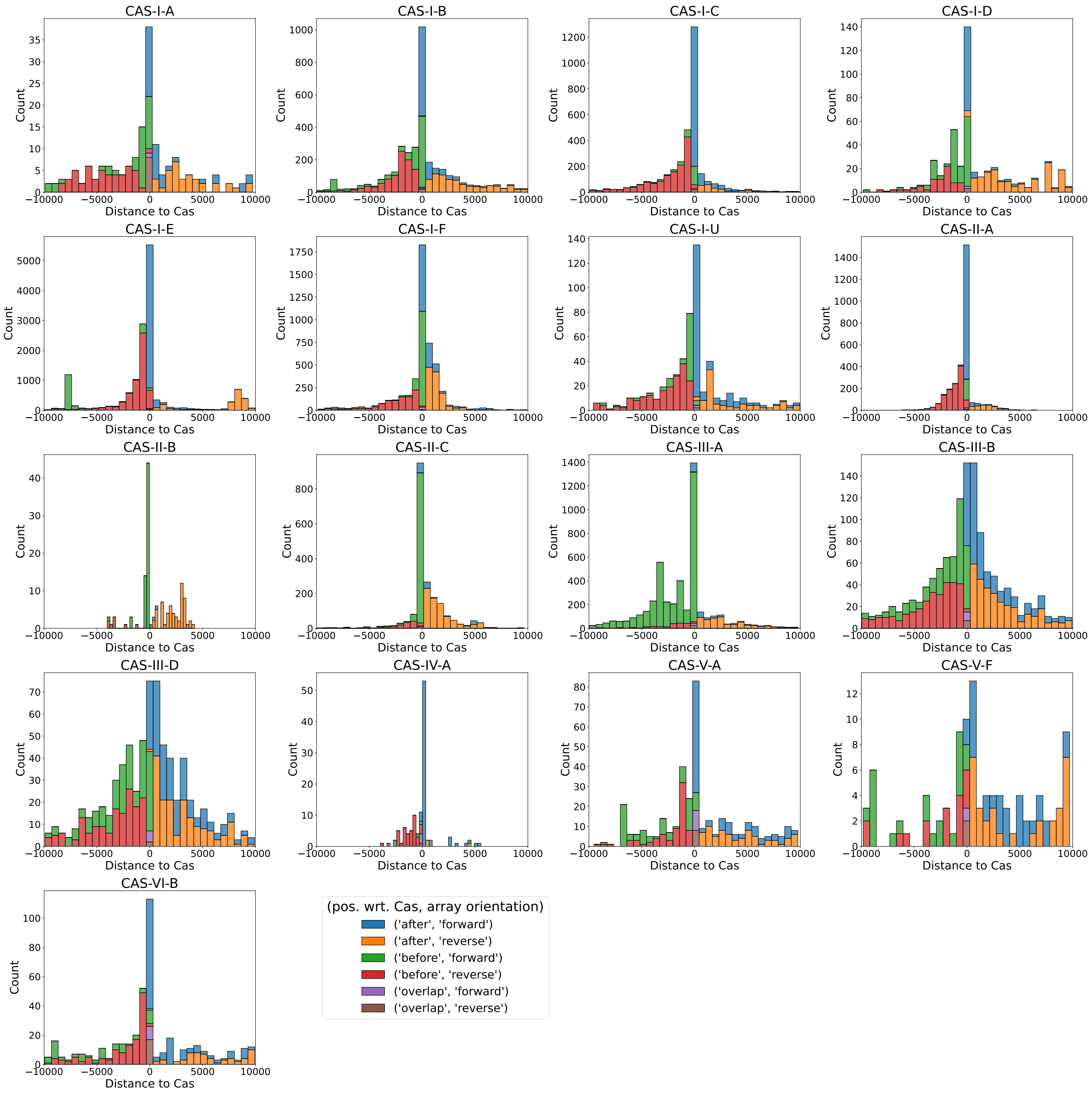

Supplementary Fig. S8. Directional distances of CRISPR arrays to their respective Cas locus. We show directional distances from the CRISPR arrays with maximum distance of 10000 bp to their respective Cas locus for all types with sufficient data. The colors show where in the genome the arrays where found and the array orientation. Green and red are 'before' the respective Cas locus (left of (and including) the 0 bin). In blue and orange are arrays 'after' the respective Cas locus (right of (and including) the 0 bin). CRISPR arrays found within the Cas locus are colored in purple and brown and naturally can only be found in the 0 bin. Green, blue and purple mark forward oriented arrays and red, orange and purple mark reverse oriented arrays. Overall, all types favor closeness to the Cas locus but we identify 5 different distribution patterns described in the main manuscript.

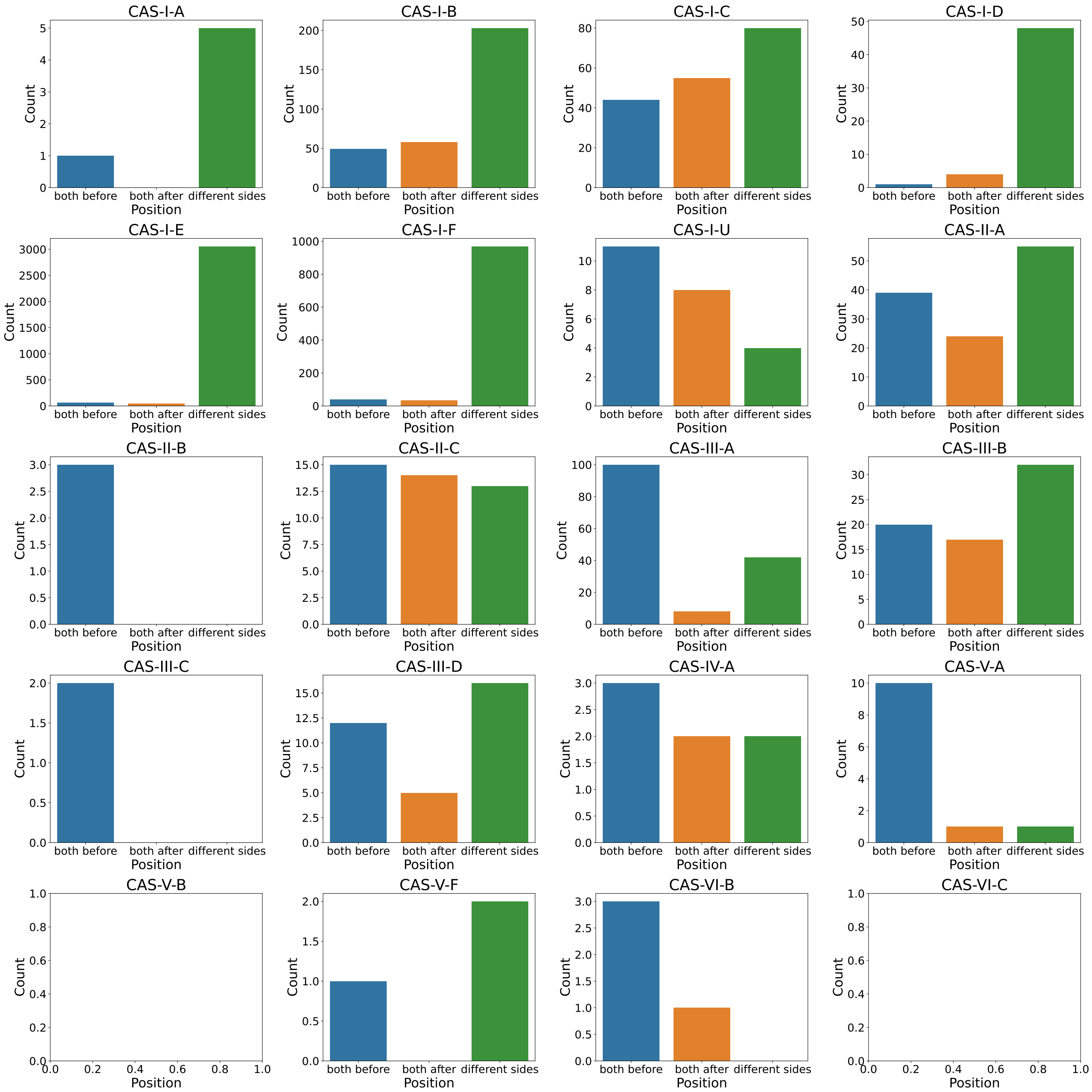

Supplementary Fig. S9. Positioning of arrays for genomes with exactly two close (< 10000 bp distance) arrays belonging to the same Cas locus. Type I-D, I-E and I-F greatly favor placing the arrays divided on both sides of the array. Other types are much more diverse in the positioning of the arrays. Note the different y-axis scales for each plot. There is not sufficient data for all types.

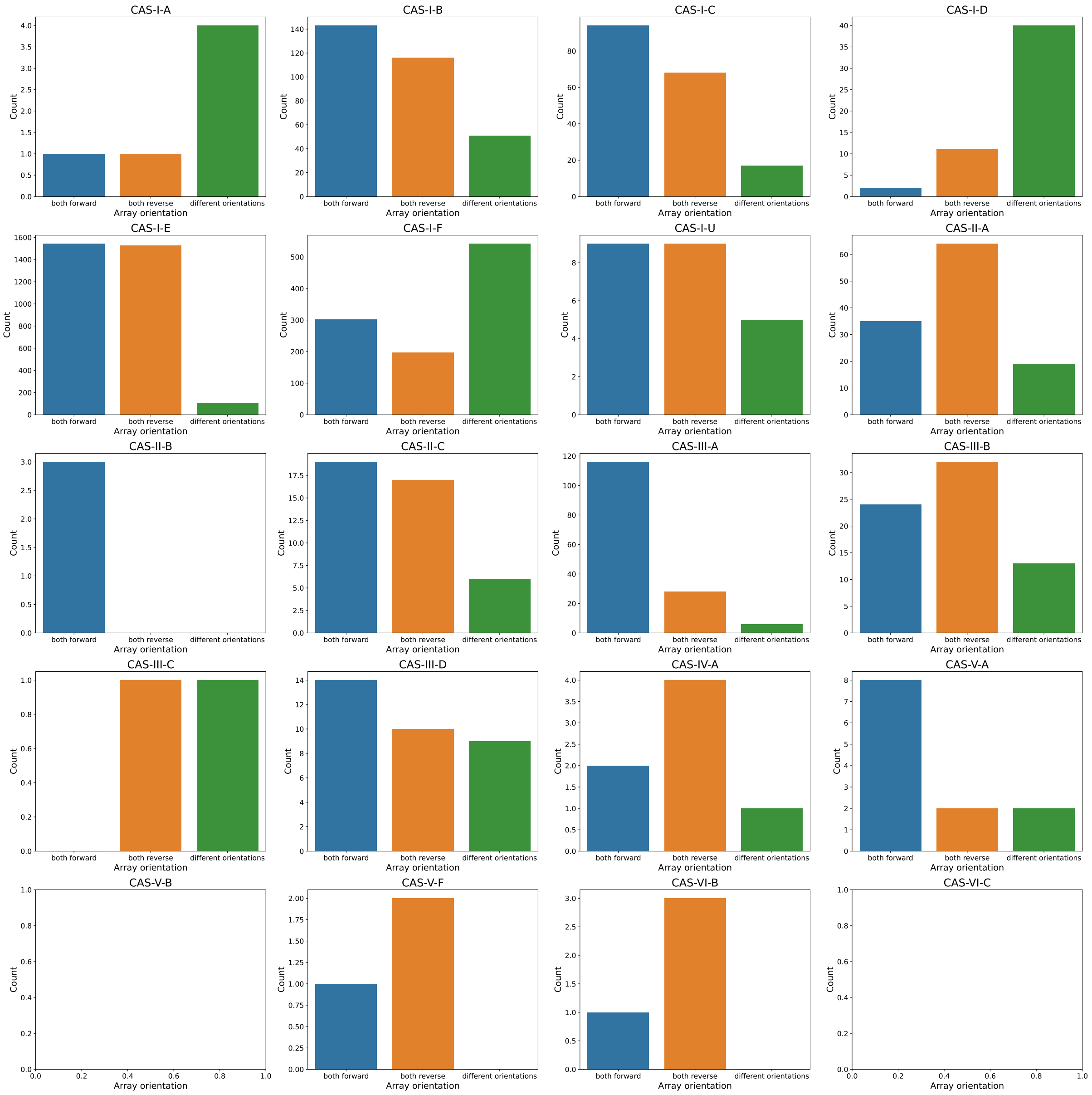

Supplementary Fig. S10. Orientation of arrays for genomes with exactly two close (< 10000 bp distance) arrays belonging to the same Cas locus. Note the great differences of distributions of the array orientation between the types I-D, I-E and I-F which often have arrays placed on different sides of the Cas locus, see Figure S9.

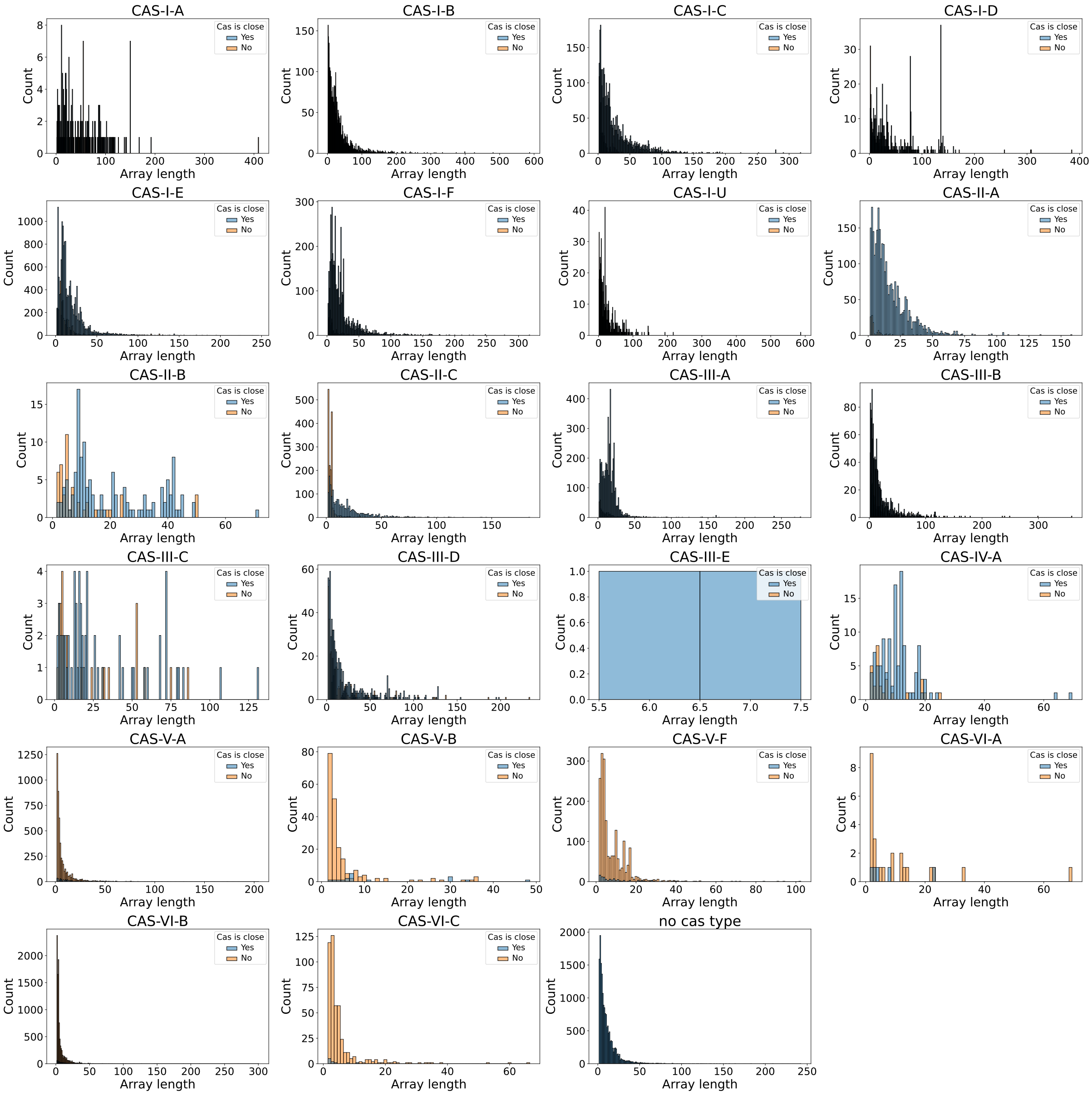

Supplementary Fig. S11. Spacer array length distributions across Cas types. We show the distribution of the number of spacers across Cas types with information about the closeness of their Cas locus. The distributions of 'Yes'/'No' for 'Cas is close' are layered over each other. In general, arrays are shorter, if they are further removed from their Cas and there are large differences between types.

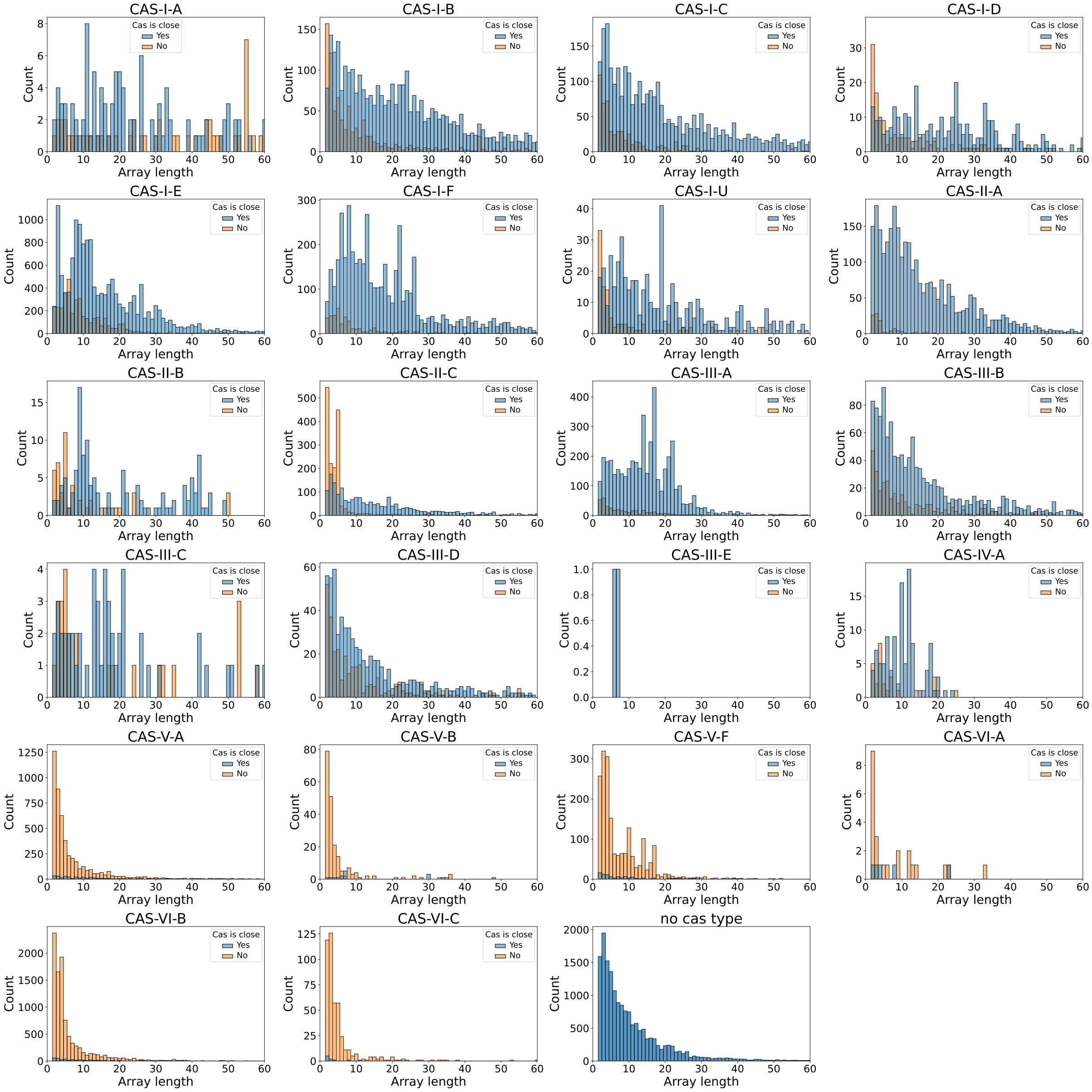

Supplementary Fig. S12. Spacer array length distributions across Cas types with limited x-axis to 60. Zoomed in version of the array length distribution plot above.

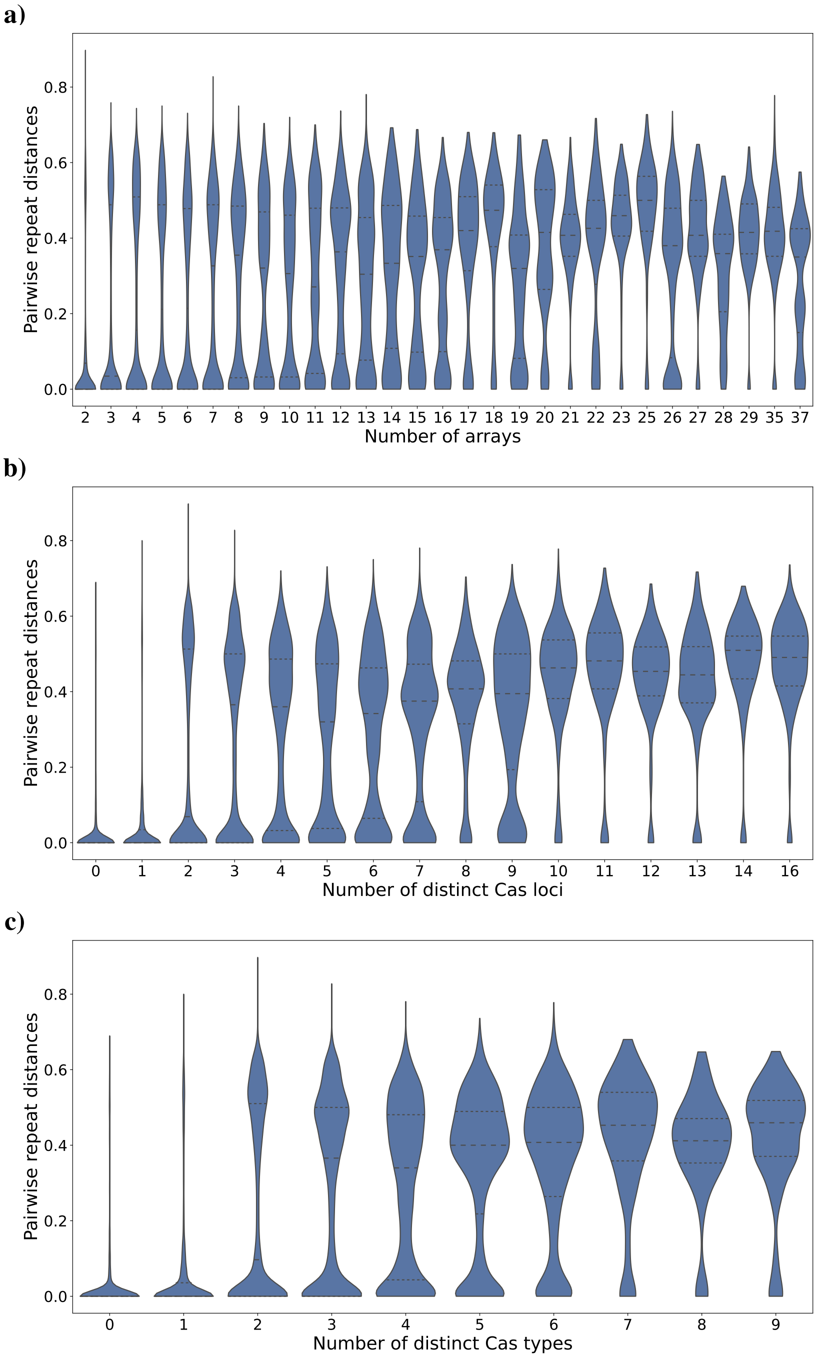

Supplementary Fig. S13. Distribution of pairwise repeat distances. We show the distribution of pairwise (normalized) repeat distances with respect to the number of arrays (**a)**), the number of distinct Cas loci within a genome (**b)**) and the number of distinct Cas types within a genome (**c)**). Violins are normalized for each column. It clearly shows that for more arrays or types the distribution becomes bi-or multimodal with peaks around 0, i.e. closely related repeats of interacting arrays (likely of the same Cas type), and higher values (0.4 - 0.5) showing the distances between arrays of (likely) different Cas types. Naturally since we compute pairwise distances the peak in 0 tends to reduce again and genomes carry decent variety of types and repeats leading to multimodal distributions.

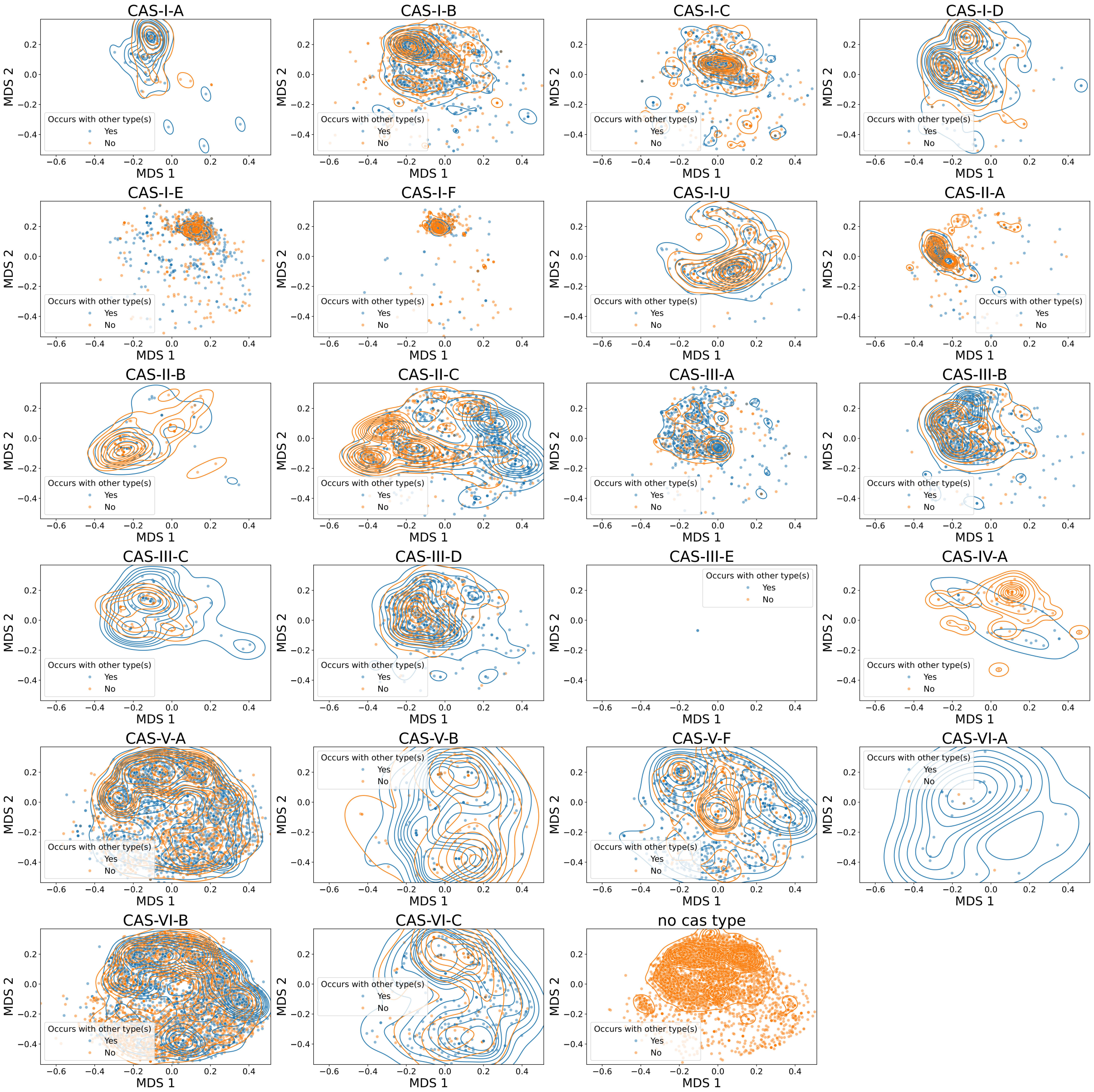

Supplementary Fig. S14. Repeat MDS into 2-d for all found Cas types (colored by: 'occurs with other type(s)'). Complementary figure to Fig. 4 in the main manuscript showing the repeat MDS for all types in the dataset with respective contours of the kernel density estimates.

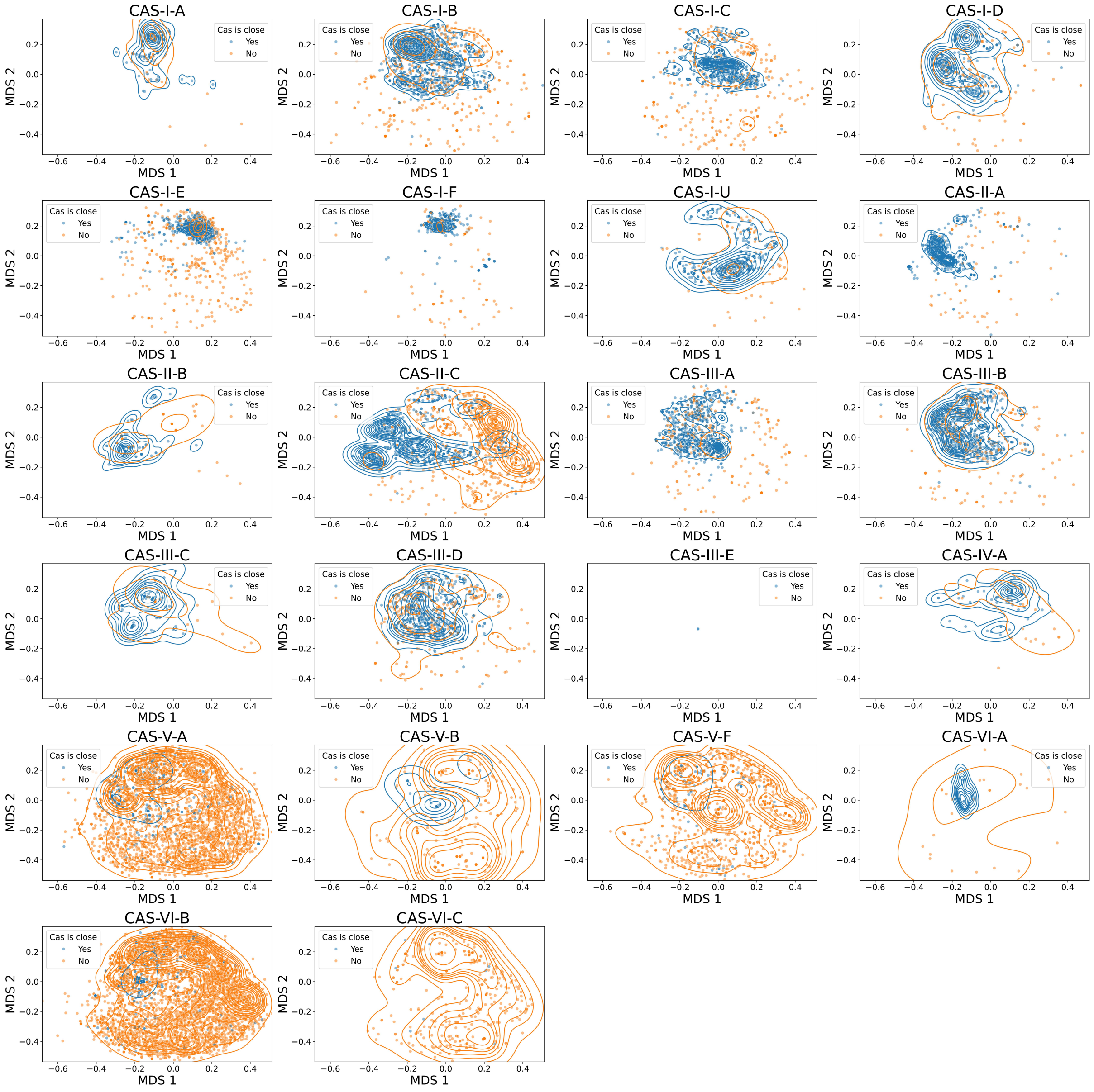

Supplementary Fig. S15. Repeat MDS into 2-d for all found Cas types (colored by 'Cas is close'). Complementary figure to Fig. 4 in the main manuscript showing the repeat MDS for all types and colored by closeness to the respective Cas locus (close if distance < 10000 bp) with respective contours of the kernel density estimates.
